# Synthesis of Long Alkyl and Fluoroalkyl Substituted Siloxane Copolymers for the Water Repellency of Rammed Earth Walls

**DOI:** 10.3390/ma17010216

**Published:** 2023-12-30

**Authors:** Keun-Byoung Yoon, Mu-Jin Kim, Dong-Eun Lee, Hee-Seon Kim

**Affiliations:** 1 Department of Polymer Science and Engineering, Kyungpook National University, Daegu 41566, Republic of Korea; kbyoon@knu.ac.kr (K.-B.Y.); anwlc9833l@gmail.com (M.-J.K.); 2 School of Architecture, Civil, Environment and Energy Engineering, Kyungpook National University, Daegu 41566, Republic of Korea; 3 Corporate R&D Center, ICAS Co., Ltd., Daegu 41566, Republic of Korea

**Keywords:** water repellency, siloxane copolymer, rammed earth wall

## Abstract

Rammed earth in a built environment has vapor diffusion characteristics and humidity control abilities, each of which is respectively attributed to the porous structures and the hydrophilic properties. Indeed, these structures and properties allow for the easy absorbance of water particles, hence affecting the durability of a rammed earth wall. This paper presents the water-repellency method for rammed earth walls, which utilizes siloxane copolymers containing fluorine. The water-repellent properties are investigated by measuring the contact angle, water absorption rate, and compressive strength after spray-coating with the synthesized siloxane copolymers on the surface of the rammed earth specimens under study. The water contact angle of the specimen, coated with a siloxane copolymer containing 10 mol.% of a silane monomer with a fluorine group, is about 140°. The water absorption of the specimen obtained after immersing in water for 24 h is low, at about 3.5 wt.%. In addition, the compressive strength remains more than 80% of the corresponding strength of the specimen which is not immersed in water. It is confirmed that the use of a 10% by volume of the siloxane copolymer containing the fluorine group may enhance the water-repellent performance and economic competitiveness.

## 1. Introduction

Rammed earth construction is used for building walls using natural raw materials, such as earth, chalk, lime, or gravel. The more the construction industry pursues sustainable natural building materials and economical construction methods, the more intensive the interest in using rammed earth construction has become in recent years. Indeed, rammed earth construction provides an easily accessible method to construct non-combustible, strong, and durable walls with excellent thermal properties [1].

However, the method encounters eroding issues when the air contact surface areas of rammed earth walls are frequently exposed to weather elements (i.e., rain, snow, fog, dew, and sleet, etc.) which continuously alternate wetting and drying. The issue causes the earth particles to retain a high amount of moisture, resulting in destructive effects [2]. The vapor diffusion properties and humidity control ability of rammed earth, which are commendable for the wall building materials, may be earned only when the permeability and hydrophilic properties of the rammed earth materials are controlled appropriately because otherwise, moisture particles can easily penetrate through the walls [2]. Indeed, the porous structure of the rammed earth wall causes high sensitivity to moisture absorption through capillary tubes in the wall. Of course, controlling water absorption is an important contribution to the reduction of various water-induced damages, such as stains, scaling attributed to freeze-thaw cycles, chemical attacks, and corrosion of the reinforcing steel [3].

Soils become solidified when they are dry but lose their strength completely when they are wet. To complement this issue, the surface treatments are used to improve the weather resistance, hence acquiring the long-term performance of the rammed earth materials. Since moisture penetration is the major factor affecting the durability of the rammed earth wall, a series of recent methods have focused on improving the durability of rammed earth structures using stabilizers, such as cement [4]. Soil stabilization binds soil particles together, improves soil strength, and increases softening resistance to water using waterproofing [5]. It is noteworthy that cement is a hydrophilic material, so it does not help water penetration, nor solve eco-friendliness, because it allows damage to the insulation of the rammed earth buildings.

The recent representative methods, which improve the durability of rammed earth buildings against natural weathering, are twofold, internal admixtures treatment and post-surface treatment [6]. The outermost surface of the rammed earth wall undergoes extreme wear and tear. The surface must endure the weathering that the external climate (i.e., sun, wind, rain, snow, ice, etc.) throws at the wall over its service life. Silicone-based repellents are useful for rammed earth constructions against existing post-surface treatments, because the sealer penetrates the capillary system and reacts with the substrate via strong bonding, hence providing long-term protection against natural weathering. The post-surface treatment using water-repellents has been well accepted as a reliable long-lasting solution to minimize water penetration within inorganic construction materials [6]. In the last decades, silane, siloxane, or silicone resin-based emulsions or powders have been used as water-repellents in mortar and concrete [6,7]. Indeed, treatment using these compounds is now an established technology with well-accepted benefits [8,9] However, these compounds have been rarely used for rammed earth walls. The dirty secret of rammed earth is that the rammed earth walls built by incorporating some kind of stabilizers do not lend themselves to providing an admissible method for long-term water control (i.e., water barrier). Note that the surface of the rammed earth wall, which is protected by existing post-surface treatments, is disarmed when any scratch and/or defect is incurred in the surface layer.

Siloxane polymer-based repellents offer permanent water-repellent layers on various types of construction materials, including soil, aggregate, and inorganic [7,10]. The reaction leads to the formation of a monolayer on the surfaces by converting the water-like silanol groups to water-repellent siloxane bonds. These siloxane polymers react with soil particles, hence forming hydrophobic layers on the surface of the soil particles.

Most siloxane-based polymer water-repellents consist of alkyl groups, such as propyl, octyl, or dodecyl groups [7,10,11]. Siloxane polymers substituted with these long-chain alkyls have a good water-repellency effect, but fluoroalkyls outperform them in hydrophobicity. Therefore, in a water-repellent study using fluoroalkyl silane, it was reported that it had an excellent water-repellent effect by dip-coating [12], mixing it with a polystyrene solution [13], or mixing it with cement [14]. However, there have been no studies on the water-repellency of using siloxane polymers substituted with fluoroalkyl on rammed earth walls, but a few studies have investigated water-repellent properties using fluoroalkyl siloxane polymers in concrete [15]. Silane compounds containing long-chain alkyls or fluoroalkyls are often used as water-repellents and are also applied to building materials. However, most of the studies have mixed silane compounds with cement or stabilizers, or coated them on the surface. No studies have been reported to consider the water-repellency performance of a siloxane copolymer containing fluoroalkyl when directly coating the rammed earth surface.

This study develops a water repellent which may increase the durability of rammed earth walls by copolymerizing various silane monomers. The water-repellent was prepared by the hydrolysis and condensation polymerization of trialkoxysilane monomers substituted with an alkyl group or a fluoroalkyl group. The performance of the water repellent, according to the type of monomer and the concentration of the siloxane copolymer solution, was investigated. This study is characterized by synthesizing a siloxane copolymer containing a small amount of fluoroalkyl and spray-coating it on a rammed earth specimen to evaluate its water-repellency performance and durability.

A low molecular weight siloxane copolymer was produced for surface spray-coating, and the water-repellent characteristics were confirmed by the type of fluoroalkyl silane monomer constituting the copolymer, and the contact angle depending on the copolymer solution concentration. Since the rammed earth wall is vulnerable to moisture, the entire surface of the specimen spray-coated with siloxane copolymer was immersed in water for 24 h, and the compressive strength of the specimen was measured in an undried state to evaluate its durability. This study characterizes a method for maximizing water-repellency performance and durability by applying a siloxane copolymer containing the smallest possible amount of fluoroalkyl to rammed earth.

## 2. Experimental

### 2.1. Materials

n-Octyltriethoxysilane (OTES, Aldrich Co., Milwaukee, WI, USA), *1H,1H,2H,2H*-perfluorodecyltriethoxy-silane (PFTES, Aldrich Co., Milwaukee, WI, USA), (3,3,3-trifluoropropyl) trimethoxysilane (TFTMS, Thermo Scientific, Waltham, MA, USA), and tetraethoxysilane (TEOS, Aldrich Co., Milwaukee, WI, USA) are used without purification for the synthesis of siloxane copolymers. For comparison with synthesized siloxane copolymers, a water-repellent from Masonry Defender (Richmond, IN, USA) and an epoxy-containing repellent from Kukdo Chemical (Seoul, Republic of Korea) are used.

### 2.2. Synthesis of Siloxane Copolymers for Water-Repellents

**Figure 1:**
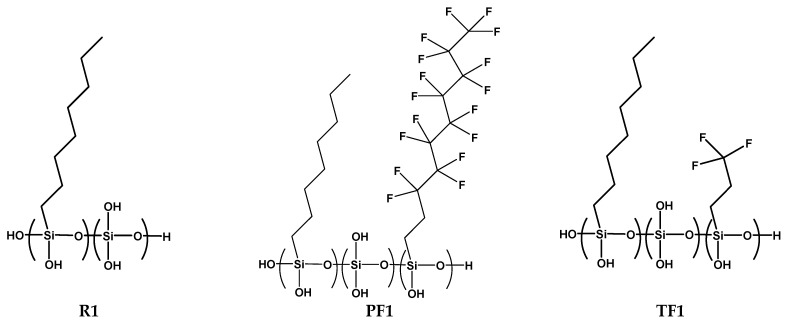
The chemical structure of siloxane copolymers is obtained by hydrolysis and condensation reactions.

Siloxane copolymers are synthesized using sol–gel hydrolysis and a partial condensation reaction of various silane monomers containing long alkyl and fluoroalkyl groups. The alkoxy group of the silane monomer is hydrolyzed under acidic aqueous conditions to produce a hydroxyl group (-OH), thereby polymerizing the hydroxyl group and the alkoxy group through a partial condensation reaction [16,17]. The siloxane copolymers are synthesized from OTES, PFTES, TFTMS, and TEOS, and ethanol is used as a co-solvent at a molar ratio of ethanol:silane 1.3:1, to ensure a miscible solution of the silane precursors. A relatively high quantity of HCL (0.5 mol/L) is used as a catalyst in the HCl, silane molar ratio of 0.01:1, to improve the stabilization in an aqueous medium. The mixture is stirred at 80 °C for 6 h, to obtain a clear and colorless siloxane copolymer solution, and the chemical structure of the obtained siloxane copolymers is shown in Figure 1.

### 2.3. Preparations of Specimens

Rammed earth specimens are prepared to measure the contact angles and the compressive strength by using cylindrical molds of which the height and the diameter are 950 and 50 mm, respectively, taking the approach of a previous study [1]. In the previous study, to prepare a rammed earth specimen a mixture was prepared by mixing 500 g of soil with 60 g of water, 40 g of a synthesized acrylic acid-acrylamide copolymer (5 wt%) solution, and 33 g of a commercial epoxy as a stabilizer [1]. After pouring the mixture into the cylindrical molds, compaction is applied using a mechanical rammer exerting an impact force of ~20 kN. Each specimen is made by placing four compaction layers sequentially. Each layer is poured with an equal amount of soil mixture. Each layer is cast in the mold in three consecutive iterations of compaction using the mechanical rammer, hence producing the specimen.

The water-repellents of 5 to 50 vol.% ethanol/water solution are produced. After spray-coating each repellent on the surface of the rammed earth specimens, the specimens are cured using natural drying, followed by 12 h drying in an 80 °C oven. It takes more than two weeks for the natural drying to arrive at the full maturity of curing. Therefore, some specimens are dried in an oven at 80 °C for 12 h to quantify the difference between the contact angles obtained using the oven-drying method and those obtained using the natural drying method. The difference in contact angle is insignificant within 2°, so curing for the experiments is carried out using oven drying, not natural drying.

### 2.4. Characterizations

The viscosity of the synthesized siloxane copolymer is measured with a Brookfield viscometer equipped with an Entered UL Adapter after removal of the solvent used for polymerization. The copolymer from which the solvent was removed under vacuum was present as a viscous clear solution, and gelation was not observed. Next, ethanol (8.5 mL) and DI water (2.5 mL) used in the polymerization were added to the copolymer from which the solvent was removed in the same amount to obtain a clear solution. (Polymerization conditions: total monomer (30 mL), ethanol (8.5 mL), and DI water (2.5 mL)).

The contact angle is measured using the sessile-drop method using a contact-angle goniometer (SEO Ind., Pheonix-150/Tilting, Suwon, Republic of Korea). The angle value is obtained by averaging at least five measurement points from various parts of each sample. Each angle point is measured given identical droplets of distilled water, the volume of which is 8 μL at room temperature.

The durability evaluation is performed by spray-coating the entire surface of the specimen twice with a siloxane copolymer, impregnating it with water for 24 h, and measuring the compressive strength without drying. Each siloxane copolymer was spray-coated by preparing a 5 to 50 wt.% solution. Then, the identical amount of solution was coated on each specimen evenly by rotating the surface at the same speed. Given the specimen has the predefined dimensions, 0.32 g of the polymer is spray-coated over the surface of the specimen when 50 wt.% solution is applied. The thickness of the functionalized layer obtained by the spray-coating, which is the spray-deposition approach, is not accurately measurable because the surface is irregular. However, it is found that the thickness is at the level of the subnanometer when coated twice. The strength is measured by performing the unconfined compression test, which is the approach of ASTM D 2166 [18] and KS F 2314 [19]. The specimens are pressed with a loading rate of 1 mm/min by moving upwards a bottom plate while recording the compression distance and stress. The values of the compressive strength are obtained by averaging three specimens with an error of less than 5%.

## 3. Results and Discussions

Siloxane polymers consist of hydrophilic and hydrophobic groups. The active group Si-OH, which is a hydrophilic group, easily reacts with the surface of the rammed earth walls, hence forming a chemically bonded layer. The outside of such a coat is composed of a water-repellent group, which consists of alkyl groups. This study involved the synthesis of siloxane copolymers, which are specialized to coat the surface of rammed earth walls. A silane with an octyl group, which is commonly used as a water-repellent, and a silane monomer in which a perfluorodecyl group and a trifluoro propyl group are substituted, were copolymerized. The copolymerization outputs obtained by performing the hydrolysis and partial condensation [20] of four types of monomers: OTES, PFTES, TFTMS, and TEOS, are shown in Table 1. A 10 mol% of TEOS was added to the blends in all polymerizations to facilitate the sol–gel hydrolysis and the condensation reaction. As shown in Figure 1 and Table 1, 10 mol% of TEOS was added to OTES in the case of R1.

The viscosity of the OTES/TEOS copolymer (R1), which is a water-repellent synthesized with OTES and TEOS in a 9/1 mol ratio, is 1300 cP, the highest of the other copolymers. The viscosity of RF1 and TF1 with fluoroalkyl-substituted PFTES and TFTMS was relatively low compared to R1 at 1000 and 800 cP. The synthesized siloxane copolymer was considered to be advantageous as a water-repellent for spray-coating, due to its low viscosity.

**Figure 2:**
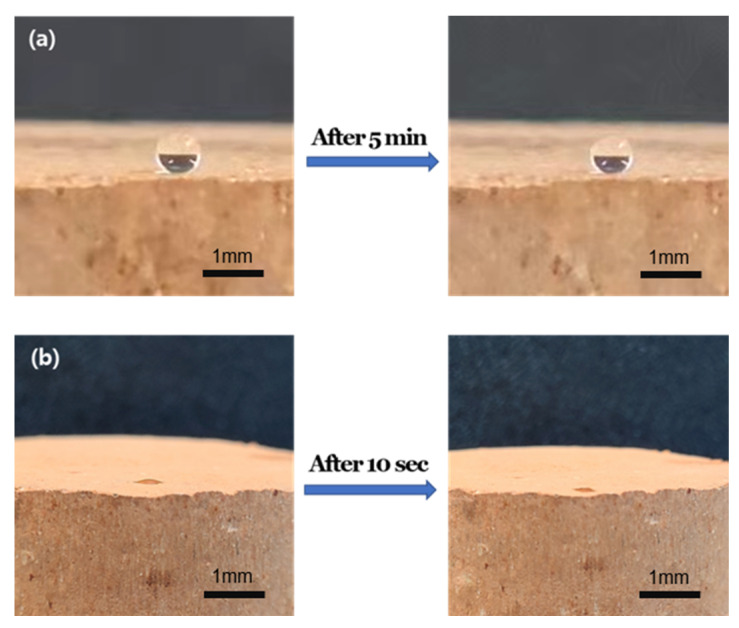
Digital photographs of water droplets on the surface of rammed earth specimens, (**a**) siloxane copolymer PF1 coated surface and (**b**) uncoated surface.

The naturally dried rammed earth specimen was spray-coated with a siloxane copolymer solution and dried, to measure the water contact angle to evaluate the water repellency performance. Figure 2 is a photograph of water droplets being dropped on a specimen coated with the siloxane copolymer PF1 and an uncoated specimen before measuring the contact angle.

As shown in Figure 2a, the droplet shape of the PF1-coated specimen was maintained 5 min after dropping the droplet, but the uncoated specimen was absorbed on the surface as soon as the droplet was dropped, and the contact angle could not be measured. Therefore, the contact angle of the siloxane copolymer-coated specimen was measured 5 s after dropping the water droplet.

**Figure 3:**
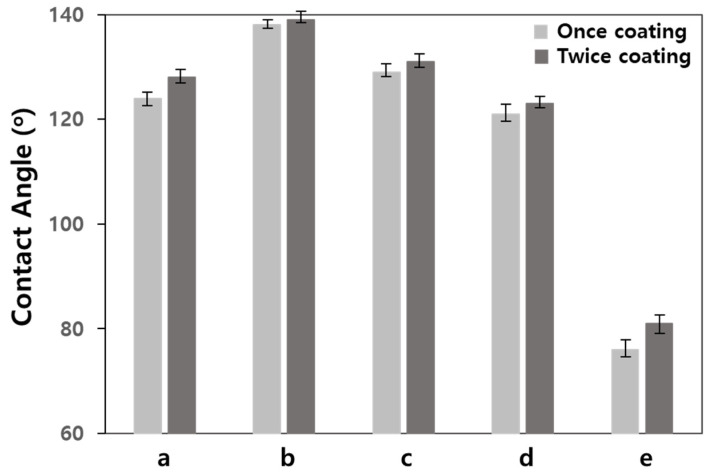
Water contact angles of the rammed earth specimens coated with the synthesized siloxane copolymers and commercial water-repellents; (a) R1, (b) PF1, (c) TF1, (d) commercial Masonry Defender, and (e) commercial epoxy repellent.

The siloxane copolymers substituted with alkyl and fluoroalkyl were spray-coated once, and twice, on the specimen, respectively, to measure the contact angle, as shown in Figure 3, and the commercial water-repellents were also measured in the same method and compared with the synthesized copolymers.

The contact angle of the water-repellent using the siloxane copolymer substituted with the fluoroalkyl group was higher than that of the siloxane copolymer substituted with the alkyl group and was superior to that of the commercially available water-repellent. In addition, regardless of the type of water-repellent, the contact angle of the twice-coated specimen was approximately 2–3° higher than that of the once-coated specimen. Because the silane-based and epoxy-based water-repellents are commercially available, they were compared with the siloxane copolymer synthesized in this study, and evaluated by their contact angle.

The PF1 siloxane copolymer containing 10 mol% of the perfluoroalkyl silane had the highest contact angle, which is considered to be due to the alkyl chain length and fluorine contents. The contact angle of the copolymer TF1 containing 10 mol% of the trifluoromethyl group was slightly higher than that of copolymer R1 composed of a long-chain alkyl. This is considered to be because the hydrophobicity of C-F is much higher than that of C-H, due to the influence of the fluorine content [21]. In addition, PF1, with a high fluorine content and a long fluoroalkyl chain, showed better water-repellency performance than TF1. These results suggest that the fluoroalkyl used for PF1 and TF1 was influenced by the fluorine content and alkyl length. Yadav et al. described, in a molecular dynamic study of self-assisted monolayers, that surface hydrophobicity increases when the chain length is long, due to the influence of chain flexibility [21]. It can be seen that siloxane copolymers with long alkyl and fluoroalkyl groups are promising for increasing the water repellency of the rammed earth wall. In general, polysiloxane containing PDMS is accepted as a representative biocompatible polymer, and the fluoroalkyl group substituted for the siloxane copolymer is more stable than the alkyl group. The C-F bond strength and C-H bond strength are 130 and 104 kcal/mol, respectively.

**Figure 4:**
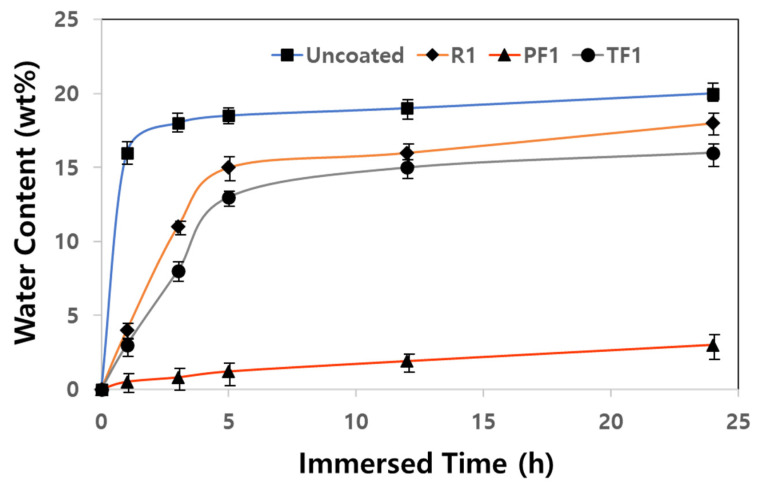
Water absorption according to immersion time of the specimens coated with siloxane copolymers and the uncoated specimen.

In particular, rammed earth buildings are rapidly reduced in durability due to moisture absorption. The immersion test is widely used for the durability evaluation of building materials using natural materials [22]. The water resistance of the siloxane copolymer was evaluated by measuring the amount of water absorption over time after immersing the rammed earth specimen coated with the siloxane copolymer and the uncoated specimen in water. After a predefined time, the specimen was taken out of the water, the surface moisture was quickly removed with a towel, and the specimen was weighed. The weight increase is indicated by the wt.% of water absorption against the initial dry weight. Figure 4 shows the increase in the water absorption amount of the specimens according to the water immersion time.

**Figure 5:**
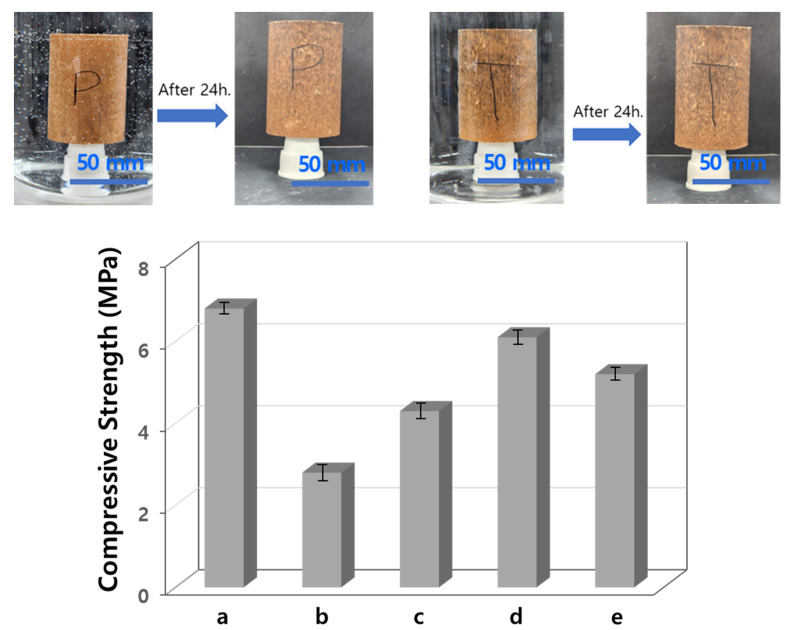
The compressive strength of a rammed earth specimen immersed in water for 24 h; (a) bare specimen, (b) immersed bare specimen, (c) a specimen coated and immersed with R1 copolymer, (d) a specimen coated and immersed with PF1 copolymer, and (e) a specimen coated and immersed with TF1 copolymer.

The specimen that is not coated with the copolymer absorbs about 18 wt.% of water after immersing in water for 1 h. The water absorption becomes almost constant, at about 20 wt.%, after immersing in water for 3 h. When the specimen was immersed in water for 5 h, there was no change in the surface of the specimen, but it was observed that a very small amount of soil particles fell at the edge. Even if a specimen that is not coated with a water-repellent is immersed for a long time, the soil particles are not completely released. The reason is because the two-component polymer stabilized strongly surrounds the soil particles, as found in the results of previous studies [1]. However, the specimen coated with the copolymer PF1 with the highest contact angle had a very low absorption of 3.5 wt.% even if immersed for 24 h. On the other hand, the specimen coated with the copolymer TF1, with a slightly lower contact angle than PF1, had a water absorption of 14 wt.% after 5 h of immersion. Even in the digital photographs of Figure 5, the deformation of specimens could not be observed when the specimens coated with the PF1 and the TF1 copolymer were immersed in water for 24 h. The difference in contact angle between PF1 and TF1, which are siloxane copolymers with a similar viscosity, was significantly different within 10°, but the water absorption rate during immersion was significantly different. This is considered to be affected by the penetration depth of the siloxane copolymer solution on the surface of the specimen, but there was a limit to identifying the cause because accurate measurement was not possible.

The compressive strengths (CSs) of the specimens used for the water absorption experiment are shown in Figure 5. Note that the specimens are coated with a water repellent developed to complement the vulnerable hydrophilicity of rammed earth walls exposed to rainfall.

The CS of the bare specimen was 6.7 MPa, and the CS of the specimen immersed for 24 h decreased sharply to 2.5 MPa. However, the CS of the in the specimen coated with PF1 having low water absorption was 6.1 MPa. It is found that the CS was hardly reduced compared to the bare specimen. After immersion, the CSs of the specimens coated with R1 and TF1, with relatively high water absorption, were 4.1 MPa and 5.1 MPa, respectively. The CSs before immersion of the specimens coated with R1, RF1, and TF1, with excellent water repellency, were 6.6, 6.5, and 6.6 MPa, and the CSs after immersion of these specimens decreased by about 40, 7, and 23%, respectively. Generally, 5 MPa or more of CS is sufficient for application to a rammed earth wall, so the specimen coated with RF1 had sufficient CS to be used, even when absorbing moisture. TF1-coated specimens with a CS of 5.1 MPa absorbed four times as much water as PF1, hence they may be more vulnerable to moisture-induced climates. A specimen without a water-repellent is not sufficient for the application of a rigid rammed earth wall, due to a sharp decrease in the CS due to water absorption. This series of experiments indicated that the developed siloxane-based copolymer containing fluorine had sufficient efficacy as a water repellent, and contributed to maintaining the durability of the wall.

**Figure 6:**
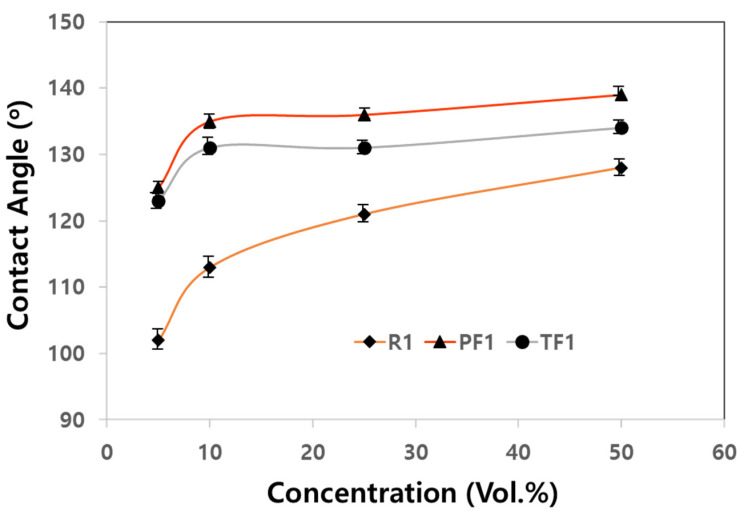
The contact angles of the rammed earth specimens according to the concentrations of R1, PF1, and TF1.

Even if only 10 mol% of a silane monomer with a high-priced fluoroalkyl group is used, the price competitiveness of either PF1 or TF1 is disadvantageous compared to a water-repellent consisting of a silane containing a conventional alkyl group. Therefore, the water-repellent effect according to the concentration of the siloxane copolymer solution was investigated. Siloxane copolymers (PF1 and TF1) containing 10 mol% of fluoroalkyl silane monomers were diluted in water to 5 to 50 vol.%, and the contact angle was measured after spray-coating (Figure 6).

The contact angles of the specimens coated with PF1 and TF1 were maintained at 135° and 130° without significant reduction even when the concentration was only 10 vol.%. However, as the concentration of the siloxane copolymers decreased the contact angle of the specimen coated with R1 that did not contain fluorine decreased sharply, and the contact angle was 112° at a concentration of 10 vol.%.

It was found that the water-repellent effect was sufficient even if only 10 vol.% of the siloxane copolymers containing a fluorine group were used, but the siloxane copolymer having a long alkyl chain without a fluorine group showed a lower contact angle even when 50 vol.% was used. Therefore, when applying a siloxane copolymer containing a fluorine group, even if the amount of the copolymer is 1/5 of that of a siloxane copolymer without a fluorine group, it is considered that it will be able to compete in price-competitiveness because of its high water-repellency performance.

## 4. Conclusions

The water-repellent which was developed in this study enhances water repellency on the surface of rammed earth walls. The repellent is obtained by copolymerizing a silane monomer substituted with a highly hydrophobic fluoroalkyl group and a conventional alkyl silane by hydrolysis and condensation. The contact angle of the water droplet on the surface of the rammed earth specimen, which is coated with the siloxane copolymer containing 10 mol.% of the siloxane monomer with a fluorine group, is about 140°. Less than 3.5% by weight of water is absorbed when this specimen is immersed in water for 24 h. The compressive strength of the specimen immersed remains 80% or more of it that is not immersed. The contact angle of the specimen which is coated with a solution produced by 10 vol.% of a siloxane copolymer containing a small amount of a fluorine group is 130° or more, hence being excellent in water repellency. The post-treatment water-repellent keeps the surface of the rammed earth walls dry, reduces water absorption, and prevents liquefaction, hence delivering admissible performance accommodating various applications. The main contribution of this study is that the deep penetration into the capillary system, which may be earned by the silicone-based repellents, allows for the natural exposure of a rammed earth wall in the long term, hence keeping intact the aesthetics of the exterior wall finish. This study has several limitations, as follows: First, it would be commendable to quantify the long-term durability of the surface that is stabilized with silicone-based repellents when experiencing repetitive extreme climate changes, such as wet and dry, freeze and thaw, and hot and cold cycles, for future study. It may contribute to establishing an ideal envelope for exterior wythes on the rammed earth wall by allowing a multiple-layer build-up. Second, this study investigated the structure of siloxane monomers containing fluorine, and the effect of fluorine on the properties of water-repellents. However, it has a limitation in that it did not conduct detailed identification of the microstructure of the siloxane copolymers, consisting of the actual composition of the coating layers by assessing with NMR. It would be desirable to identify the water-repellent properties according to the structure of various fluorine-containing siloxane monomers.

## Figures and Tables

**Table 1:** The viscosity of copolymers at different monomer feed ratios.

	Monomer Feed Ratio [OTES]/[PFTES]/[TFTMS]/[TEOS]	Viscosity (cP)
R1	9/0/0/1	1300
PF1	8/1/0/1	1000
TF1	8/0/1/1	800

Copolymerization condition: [Monomer]/[EtOH] = 1/1.3, [Monomer]/[H_2_O] = 1/10, [Monomer]/[HCl] = 1/0.01, 80 °C, 6 h.

## Data Availability

Data will be provided upon request.
